# Supplementary material for: The Mesozoic terminated in boreal spring
Source: Nature. 2022 Feb 23;603(7899):91–4. doi: 10.1038/s41586-022-04446-1 (PMC8891016; doi:10.1038/s41586-022-04446-1)
Supplement: Supplementary file 1 — Reporting Summary [file 41586_2022_4446_MOESM1_ESM.pdf]

nature portfolio

Corresponding author(s): Melanie During

Last updated by author(s): December 16, 2021

Reporting Summary

Nature Portfolio wishes to improve the reproducibility of the work that we publish. This form provides structure for consistency and transparency in reporting. For further information on Nature Portfolio policies, see our Editorial Policies and the Editorial Policy Checklist.

Statistics

For all statistical analyses, confirm that the following items are present in the figure legend, table legend, main text, or Methods section.

n/a	Confirmed	
☐	☒	The exact sample size (*n*) for each experimental group/condition, given as a discrete number and unit of measurement
☐	☒	A statement on whether measurements were taken from distinct samples or whether the same sample was measured repeatedly
☒	☐	The statistical test(s) used AND whether they are one- or two-sided<br>*Only common tests should be described solely by name; describe more complex techniques in the Methods section.*
☒	☐	A description of all covariates tested
☒	☐	A description of any assumptions or corrections, such as tests of normality and adjustment for multiple comparisons
☐	☒	A full description of the statistical parameters including central tendency (e.g. means) or other basic estimates (e.g. regression coefficient) AND variation (e.g. standard deviation) or associated estimates of uncertainty (e.g. confidence intervals)
☒	☐	For null hypothesis testing, the test statistic (e.g. *F*, *t*, *r*) with confidence intervals, effect sizes, degrees of freedom and *P* value noted<br>*Give P values as exact values whenever suitable.*
☒	☐	For Bayesian analysis, information on the choice of priors and Markov chain Monte Carlo settings
☒	☐	For hierarchical and complex designs, identification of the appropriate level for tests and full reporting of outcomes
☒	☐	Estimates of effect sizes (e.g. Cohen's *d*, Pearson's *r*), indicating how they were calculated

Our web collection on statistics for biologists contains articles on many of the points above.

Software and code

Policy information about availability of computer code

Data collection	Inhouse ESRF MATLAB (version 2017a) scripts developed for vertical concatanation, conversion, and ring artefact corrections.
Data analysis	ESRF software PyHST2, VGStudio Max 3.2

For manuscripts utilizing custom algorithms or software that are central to the research but not yet described in published literature, software must be made available to editors and reviewers. We strongly encourage code deposition in a community repository (e.g. GitHub). See the Nature Portfolio guidelines for submitting code & software for further information.

Data

Policy information about availability of data

All manuscripts must include a data availability statement. This statement should provide the following information, where applicable:

- Accession codes, unique identifiers, or web links for publicly available datasets
- A description of any restrictions on data availability
- For clinical datasets or third party data, please ensure that the statement adheres to our policy

All scanning data are available at https://doi.org/10.5281/zenodo.5776294 and the http://paleo.esrf.eu database.

Field-specific reporting

Please select the one below that is the best fit for your research. If you are not sure, read the appropriate sections before making your selection.

☐ Life sciences ☐ Behavioural & social sciences ☒ Ecological, evolutionary & environmental sciences

For a reference copy of the document with all sections, see nature.com/documents/nr-reporting-summary-flat.pdf

Ecological, evolutionary & environmental sciences study design

All studies must disclose on these points even when the disclosure is negative.

Study description

Osteohistology of six fossil fishes, that died on the day of Chicxulub impact, representing two acipenserifom taxa is combined with isotope analyses of one of these specimens to reveal the seasonality of the last years of the Mesozoic and the season of the impact. Another specimen of acipenseriform fish was furthermore scanned to confirm that these fishes had died because of the accumulation of impact spherules into the gill region at the time of death.

Research sample

Seven acipenseriform fishes of which 3 sturgon pectoral fin spines and 3 paddlefish dentaries and 1 partial paddlefish that was scanned nondestructively. The 3 sturgeon pectoral fin spines and 3 paddlefish dentaries were made available to us by the Palm Beach Museum of Natural History following fieldwork in august 2017 and the partial paddlefish was later made available by the Palm Beach Museum of Natural History for synchrotron scanning. The sample is meant to represent accipenseriform fishes of North America during the latest Cretaceous.

Sampling strategy

Four out of six of the samples were excavated from the sediment matrix. These included all sturgeon pectoral fin spines (VUA.GG.2017.X-2743M, VUA.GG.2017.X-2744M, and VUA.GG.2017.MDX-3) and one of the paddlefish dentaries (VUA.GG.2017.X-2724). Paddlefish dentaries VUA.GG.2017.X-2733A and VUA.GG.2017.X-2733B were fractured upon discovery. To avoid further damage, the specimens were embedded in epoxy resin prior to thin sectioning. All specimens were cut with a diamond saw and polished to obtain microscopic thin sections (~50 µm thick) and thick sections for micro milling (~200 µm thick).
One partial paddlefish (FAU.DGS.ND.161.4559.T), provided by the Palm Beach Museum of Natural History was only scanned nondestructively

Data collection

Fragments of the paddlefish and sturgeon samples that remained after thin sectioning were analysed with Micro X-ray Fluorescence (µXRF) by M.A.D.During and K.H.W. Stein. High-resolution elemental mapping was conducted using a Bruker M4 Tornado 2D spectrometer at 50 kV and 600µA, without a filter, and at an acquisition rate of 20 µm/5 ms at the Vrije Universiteit Brussel (VUB).

The growth increments were sampled in the thick sections by M.A.D.During (~200 µm thick) at the highest possible accuracy using a Micromill (Merkantek). Drill transects were assigned in the accompanying software and after each individual sample was collected, the drill bit was cleaned with ethanol. Not all thick-sections were suitable for micromilling.

Micromilled hydroxyapatite samples of specimen VUA.GG.2017.X-2724 weighing ~50 µg were placed in Exetainer vials (Labco, Lampeter, UK) and flushed with purified helium gas by M.A.D. During. Orthophosphoric acid was subsequently added by S. Verdegaal-Warmerdam and allowed to react for 24 hours at 45°C. VUA.GG.2017.MDX-3 was routinely analysed with a Thermo Finnigan Deltaplus mass spectrometer connected to a Thermo Finnigan GasBench II at the Earth Sciences Stable Isotope Laboratory (Vrije Universiteit, Amsterdam) by S.Verdegaal-Warmerdam. However, the amount of CO2 generated was found to be too small to permit reliable isotopic determinations. To alleviate this, the GasBench was subsequently interfaced with a cold trap by S. Verdegaal-Warmerdam and J.(H)J.L.Van der Lubbe, where the CO2 was frozen with liquid nitrogen during a 2-minute period. After trapping for 2 minutes, an accurate single-pulse measurement was performed, for each of the samples and standards.

Synchrotron data acquisition took place in May 2018 on Beamline BM05 at the European Synchrotron Radiation Facility, Grenoble, France by M.A.D. During, D.F.A.E. Voeten, C. Berruyer & P. Tafforeau. FAU.DGS.ND.161.4559.T was scanned at an average energy of 132 keV using the white beam of BM05 filtered with 0.4 mm of Mo and 9 mm of Cu. The detector was composed of a 2-mm-thick LuAG:Ce scintillator optically coupled to a PCO edge 4.2 CLHS sCMOS camera. The resulting voxel size was 43.5 µm. In order to obtain sufficient propagation phase contrast, the distance between the sample and the detector was set at 5 m. A total of 205 scans, each consisting of 5000 projections taken at 7 ms intervals, were performed with a vertical displacement of 1.4 mm at a vertical field of view of 2.8 mm to ensure a double scan of the complete samples. Scans were performed in half-acquisition mode to enlarge the lateral field of view. The volume was reconstructed using single-distance phase retrieval algorithm coupled with filtered back projection as implemented in the ESRF software PyHST2. Vertical concatenation, 16-bit conversion, and ring artefact corrections were performed using MATLAB scripts developed in-house. The gill region and impact spherules were subsequently scanned at a voxel size of 13.67 µm (filters: 0.4 mm of Mo and 6 mm of Cu, scintillator: LuAG:Ce, 500 µm thick, detected energy: 166 keV, propagation distance: 2.5 m). The samples were scanned in half-acquisition mode in two columns of 77 scans, each consisting of 4998 projections with exposure times of 0.05 s, that were laterally concatenated after reconstruction.

Finally, samples (VUA.GG.2017.X-2724) from the paddlefish dentaries and (VUA.GG.2017.MDX-3, VUA.GG.2017.X-2743M and VUA.GG.2017.X-2744M) sturgeon pectoral fin spines were scanned at 4.35 µm voxel size for osteohistological analysis54 (filters: 3.5 mm of Al plus 11 bars Al with a diameter of 5 mm, scintillator: LuAG:Ce scintillator, 500 µm thick, detected energy: 92 keV, propagation distance: 1.5 m). The samples were scanned in half-acquisition mode in one single column of 22 scans, each consisting of 4998 projections with exposure times of 60 ms.

Digital 3D extraction of the bones and impact spherules was performed in VGStudio MAX 3.2 (Volume Graphics, Heidelberg, Germany) by M.A.D. During and D.F.A.E. Voeten. VGStudio MAX 3.2 furthermore enabled creation of virtual thick sections of the osteohistological samples through the ‘thick slab-mode’, which captures the maximum, average, or minimum, grey-level values along

	the desired field depth. Virtual thick sections were obtained from the average grey-level values at a thickness of 100 µm following optimal 3D alignment of the annuli and lines of arrested growth (LAGs). Additional virtual thick sections were created from the minimum grey-level values at a thickness of 200 µm to best resolve the sizes and distributions of osteocyte lacunae.
Timing and spatial scale	Specimens were obtained from the field in August (1-18) 2017 over an approximate distance of 2 square meters (Specimens were shipped with a delay due to hurricane season).<br>Thin and thick sections were cut on December 5, 2017.<br>X-Ray Fluorescence took place on March 13, 2018.<br>Stable Isotope analyses took place after 2 months of micromilling, without the cold trap on May 28, 2018 and with the cold trap in June 19, 2018.<br>Synchrotron scanning took place in May (3-5), 2018
Data exclusions	Oxygen and Carbon isotopic data for sturgeon pectoral fin spine VUA.GG.2017.MDX-3 were excluded due to the unreliability of the data. The amplitude for the measurements were deemed too small to offer reliable results as a consequence of the small sample size. The incremental micromill sample lines 1-5, 10-12 and 25 for paddlefish dentary VUA.GG.2017.X-2724 did not retrieve sufficient material for isotopic analyses (<2 V) and/or the atmospheric contamination as is indicated by the presence of a nitrogen peak, which led to their exclusion.
Reproducibility	For the isotopic analyses, the stability of the mass spectrometer was assured by the isotopic analysis of six reference gas peaks preceding each sample and standard measurement. This so-called monitor gas is routinely calibrated with carbonate standards with internationally accepted values. For linearity corrections, the inter-laboratorial apatitic standard (Ag-lox) has been measured four times within the sample run. The sample sizes that were obtained from specimen VUA.GG.2017.X-2724 do not allow for replicates. The analytical procedure and possible limitations of which are described in detail in the methods section.
Randomization	n.a. due to the nature of the available fossil material.
Blinding	n.a. due to the nature of the available fossil material.

Did the study involve field work? ☒ Yes ☐ No

Field work, collection and transport

Field conditions	Conditions in August 2017 varied from extremely hot and dry (~35 degrees Celsius) to extemely wet and roughly 20 degrees Celsius for 3 days, during which we did not excavate any material as we risked getting stuck in the mud.
Location	Tanis North Dakota: 46.031403"N, -103.796603"W
Access & import/export	Access was permitted via the Palm Beach Museum of Natural history and all specimen transactions as well.<br>The studied specimens were excavated at the Tanis site. Application for off-site shipment has been granted under number X24.4.T of access to research site. Application #: BV60717
Disturbance	Nothing was touched unless it was taken for study, no organisms (wild or agricultural were confronted or hurt)

Reporting for specific materials, systems and methods

We require information from authors about some types of materials, experimental systems and methods used in many studies. Here, indicate whether each material, system or method listed is relevant to your study. If you are not sure if a list item applies to your research, read the appropriate section before selecting a response.

Materials & experimental systems

n/a	Involved in the study	
☒	☐	Antibodies
☒	☐	Eukaryotic cell lines
☐	☒	Palaeontology and archaeology
☒	☐	Animals and other organisms
☒	☐	Human research participants
☒	☐	Clinical data
☒	☐	Dual use research of concern

Methods

n/a	Involved in the study	
☒	☐	ChIP-seq
☒	☐	Flow cytometry
☒	☐	MRI-based neuroimaging

Palaeontology and Archaeology

Specimen provenance	Tanis, North Dakota, United States of America: Site: PBMNH.ND.X17.54 Tanis. Access was permitted via the Palm Beach Museum of Natural history and all specimen transactions as well. Application for off-site shipment has been granted under number X24.4.T of access to research site. Application number: BV60717

Specimen deposition	All specimens are available at the Palm Beach Museum of Natural History and the VU Amsterdam
Dating methods	No dates are provided

☒ Tick this box to confirm that the raw and calibrated dates are available in the paper or in Supplementary Information.

Ethics oversight	n.a. all specimens are fossil.

Note that full information on the approval of the study protocol must also be provided in the manuscript.
