# Supplementary material for: The Mesozoic terminated in boreal spring
Source: Nature. 2022 Feb 23;603(7899):91–4. doi: 10.1038/s41586-022-04446-1 (PMC8891016; doi:10.1038/s41586-022-04446-1)
Supplement: Supplementary file 2 — Supplementary Data Table [file 41586_2022_4446_MOESM2_ESM.docx]

| Incremental growth distance (mm) from the periosteal surface | 𝛿^13^C_sc_ values (‰) VPDB | 𝛿^18^O_sc_ values (‰) VPDB | 𝛿^18^O_sc_ values (‰) VSMOW  𝛿^18^O(VSMOW) = 1.03091 x (𝛿^18^O VPDB) + 30.91 | Equivalent 𝛿^18^O_p_ values (‰) VSMOW based on^61^ | Estimated water temperatures (°C)^57^ assuming a water 𝛿^18^O value of -13.2 ‰ (VSMOW) based on a mean annual air temperature of 19°C^62, 63^ |
| --- | --- | --- | --- | --- | --- |
| 0.08 | -2.46 | -11.67 | 18.88 | 10.44 | 18.95 |
| 0.15 | -2.69 | -11.44 | 19.12 | 10.66 | 18.01 |
| 0.23 | -2.84 | -11.49 | 19.06 | 10.61 | 18.23 |
| 0.31 | -1.43 | -11.74 | 18.81 | 10.36 | 19.26 |
| 0.39 | -1.32 | -11.74 | 18.81 | 10.37 | 19.25 |
| 0.54 | -2.92 | -12.13 | 18.40 | 9.98 | 20.88 |
| 0.62 | -2.62 | -12.00 | 18.54 | 10.11 | 20.34 |
| 0.70 | -1.63 | -11.78 | 18.76 | 10.32 | 19.43 |
| 0.77 | -2.33 | -11.81 | 18.74 | 10.30 | 19.54 |
| 0.85 | -2.02 | -11.90 | 18.64 | 10.21 | 19.92 |
| 0.93 | -2.31 | -12.62 | 17.90 | 9.50 | 22.89 |
| 1.01 | -2.62 | -12.40 | 18.12 | 9.72 | 21.99 |
| 1.08 | -2.33 | -11.11 | 19.46 | 10.99 | 16.64 |
| 1.16 | -1.70 | -12.13 | 18.40 | 9.98 | 20.87 |
| 1.24 | -1.80 | -11.76 | 18.79 | 10.35 | 19.34 |
| 1.31 | -2.14 | -11.54 | 19.01 | 10.56 | 18.44 |
| 1.39 | -2.22 | -12.04 | 18.50 | 10.07 | 20.50 |
| 1.70 | -3.02 | -11.15 | 19.42 | 10.95 | 16.80 |
| 1.78 | -2.47 | -11.14 | 19.42 | 10.95 | 16.78 |
| 1.86 | -2.77 | -11.26 | 19.31 | 10.84 | 17.26 |
| 1.93 | -3.33 | -11.29 | 19.27 | 10.80 | 17.40 |

**Supplementary Data Table 1**

Isotopic data of paddlefish dentary VUA.GG.2017.X-2724. 𝛿^18^O_sc_ (VSMOW) variability in fish apatite was converted to 𝛿^18^O_p_ values following the equilibrium fractionation of carbonate and phosphate observed in biogenic apatite^61^. These 𝛿^18^O_p_ values permit tentative estimates of water temperature and the 𝛿^18^O value of the ambient water^57^. Assuming constant 𝛿^18^O water values, VUA.GG.2017.X-2724 registered a temperature range of 6°C, consistent with local seasonal estimates^12,14^. Conversely, assuming water temperatures close to annual mean air temperate estimates of 19°C^12,14^, an average water 𝛿^18^O value of circa -13 ‰ can be inferred for VUA.GG.2017.X-2724. This is in line with previous regional estimates for late Cretaceous rivers^62,63^. The coupled effect of temperature and 𝛿^18^O water variability hamper detailed registration of seasonality in the 𝛿^18^O_sc_ record, but does indicate that this paddlefish did not engage in seasonal migration between freshwater and more saline habitats.

References

62. Pellegrini, M., Lee-Thorp, J. A. & Donahue, R. E. Exploring the variation of the δ^18^O_p_ and δ^18^O_c_ relationship in enamel increments. Palaeogeogr. Palaeocl. 310, 71–83 (2011).

63. Pucéat, E. et al. Revised phosphate–water fractionation equation reassessing paleotemperatures derived from biogenic apatite. Earth Planet. Sci. Lett. 298, 135–142 (2010).

64. Fricke, H. C. & Pearson, D. A. Stable isotope evidence for changes in dietary niche partitioning among hadrosaurian and ceratopsian dinosaurs of the Hell Creek Formation, North Dakota. Paleobiology 34, 534–552 (2008).

65. Dettman, D. L. & Lohmann, K. C. Oxygen isotope evidence for high-altitude snow in the Laramide Rocky Mountains of North America during the Late Cretaceous and Paleogene. Geology 28, 243–246 (2000)
